# Temporal clustering of neuroblastic tumours in children and young adults from Scotland

**DOI:** 10.1186/s12940-026-01266-y

**Published:** 2026-01-24

**Authors:** Nermine Basta, Min Li, Louise Hayes, Colin R. Muirhead, Deborah A. Tweddle, Richard J.Q McNally

**Affiliations:** 1 Population Health Sciences Institute & Newcastle University Centre for Cancer, Newcastle University, Sir James Spence Institute, Royal Victoria Infirmary, Queen Victoria Road, Newcastle upon Tyne , NE1 4LP UK; 2 Population Health Sciences Institute, Newcastle University, Newcastle upon Tyne, UK; 3 Newcastle University Centre for Cancer, Newcastle University, Newcastle upon Tyne, UK; 4 Wolfson Childhood Cancer Research Centre, Translational and Clinical Research Institute Newcastle University, Newcastle upon Tyne, UK; 5 Great North Children’s Hospital, Newcastle upon Tyne, UK

**Keywords:** Epidemiology, Neuroblastic tumours, Temporal clustering

## Abstract

**Background:**

Neuroblastic tumours (neuroblastoma and ganglioneuroblastoma) are the most common childhood solid tumours outside the central nervous system, with a median age of diagnosis of 2 years. Temporal clustering of neuroblastic tumours in northern England and Ontario, Canada has been previously reported.

**Methods:**

We extracted data from the Scottish Cancer Registry to determine whether there was evidence of temporal clustering of neuroblastic tumours. Cases diagnosed in children and young adults aged 0–24 years between 2000 and 2020 were analysed. A modified version of the Potthoff-Whittinghill method was used to test for temporal clustering. Estimates of extra-Poisson variation (EPV) and standard errors (SE) were derived.

**Results:**

One hundred and sixty-one cases of neuroblastic tumours, aged 0–24 years, were diagnosed during the study period. Overall, there was statistically significant temporal clustering between years within the full study period (EPV = 9.13, SE = 0.22, *P* < 0.001). In addition, for cases aged < 18 months, there was significant temporal clustering between months within quarters (EPV = 0.77, SE = 0.41, *P* = 0.044). For cases aged 18 months – 24 years, there was significant temporal clustering between fortnights within months (EPV = 1.00, SE = 0.47, *P* = 0.012).

**Conclusions:**

The finding of temporal clustering is consistent with the involvement of one or more, as yet unknown, transient environmental agents in the aetiology of neuroblastic tumours.

## Introduction

Neuroblastic tumours, including neuroblastoma and ganglioneuroblastoma are the most common solid tumours outside the central nervous system in children, with a median age of diagnosis of 2 years in the UK [1]. Approximately 8 cases of neuroblastic tumours are diagnosed annually in Scotland [2]. Both genetic and environmental factors have been implicated in aetiology. Previous studies have highlighted a number of potential factors conferring greater risk including higher number of siblings, pesticides, ambient air toxic exposures whilst in-utero, use of certain medications by the mothers, consumption of alcohol and smoking during pregnancy. In contrast childhood infections, breast feeding, maternal fetal loss, folic acid, allergies and Down syndrome have been identified as potentially protective factors [3–14].

Temporal clustering occurs when an excess of cases is found at distinct, variable and transient points in time. There are different scenarios when this type of clustering might occur. Such clustering might be exhibited when there are a few relatively lengthy temporal periods (lasting months or years) with greatly increased incidence, or many more relatively short temporal periods (lasting months, or only a few weeks) with less markedly increased incidence. It is important to distinguish irregular temporal clustering from seasonal variation, which by contrast occurs every year at consistent times. The methods used to analyse seasonal variation are not the same as those used for temporal clustering. In the present study the methodology used is an adaptation of a test that was first described by Potthoff and Whittinghill to determine the presence of extra-Poisson variation (EPV) [4, 15, 16].

Two previous studies of ours from northern England and Ontario, Canada have found statistically significant evidence of temporal clustering amongst cases of neuroblastic tumours. The results, from both studies, suggested that a transient agent in the environment might be involved in the aetiology. The occurrence of such an agent would be geographically widespread and would exhibit a pattern of ‘minor epidemics’ [4, 17].

The aim of the present study was to test for temporal clustering of neuroblastic tumours amongst children and young adults (aged 0–24 years) who were diagnosed in Scotland during the period 2000–2020.

## Methods

All cases aged 0–24 years, diagnosed with a neuroblastic tumour (neuroblastoma or ganglioneuroblastoma) during the period 2000–2020 were extracted from the Scottish Cancer Registry (SCR) [2]. This time period was chosen as data collected then were of better quality than earlier data. Cancer cases in Scotland are registered through an integrated system that collects data from a number of different sources. These are combined to create individual case registrations based on the primary cancer type. Data are collected from pathology, radiotherapy, oncology, neuro-oncology and haematology departments. Further data are collected from national databases of hospital admissions, screening programmes, prospective cancer audits and death registries. These registries are linked by probabilistic matching and merged to create a record for each cancer diagnosis, which is linked to the admission. The population aged 0–24 years in the whole of Scotland was approximately 1.5 million (based on the 2022 census) [18].

### Prior hypothesis

The following hypothesis, related to the aetiology of neuroblastic tumours, was tested: a main factor causing temporal heterogeneity in the diagnosis of neuroblastic tumours is related to a geographically widespread, irregularly occurring environmental factor that exhibits temporal variation in strength and occurs around the time of diagnosis or at similar times before diagnosis.

### Statistical analysis

The statistical methods employed were the same as those previously used by us for analysis of temporal clustering of disease [4, 17]. An adapted version of the method originally developed by Potthoff and Whittinghill was used [15, 16]. The method was used to determine the extra-Poisson variation (EPV) in the number of neuroblastic tumour diagnoses per fortnight, month, quarter and year. The presence of EPV could indicate the presence of some unobserved or unaccounted factor that increases the variability of the data. Specifically, the number of diagnoses was assumed to follow a negative binomial distribution, where the ratio of the variance to the expected number of diagnoses was equal to a constant, denoted as 1 + β. When β is equal to zero, the distribution of the number of diagnoses was Poisson distributed. However, if β was greater than zero, the number of diagnoses exhibited an EPV, indicating that they were excessively dispersed compared to the Poisson distribution. The estimation of the EPV and its standard error was based on the score statistic [19]. The test for the EPV was a one-sided test (β > 0). *P* values were determined by 10,000 simulations assuming β = 0. Statistical significance was assessed using a critical value of *P* < 0.05. Results with *P* values between 0.05 and 0.10 were considered to provide weaker evidence of temporal clustering.

The effect of long-term variation was removed by placing a condition on the total number of cases in a given time period (i.e. January to March; April to June; July to September; October to December) when analysing short term patterns. This helps to provide a clearer view of short-term variations without the interference of changes in trends over longer time spans. Focused analyses were divided into the following situations: between halves of a month (i.e. fortnights); between months within a quarter; between quarters within a year; and between years. As the analysis between fortnights within months was conditional on the total number of cases per month, the results of this analysis were independent of the results of the analysis between each month within quarters. This means these analyses were conditional, and the interpretations of each were independent of the other analyses and did not influence each other [4, 17].

The date of diagnosis was determined by the date of pathological confirmation, which was consistently recorded. Each fortnight period was described in accordance with the previously established definition. In practice, they were the first 15 days of the calendar month (the first 14 days in February), compared to the remaining days of the month. The calculation of the expected number of cases in each period considered the variation in the length of the fortnights, months and years. It was assumed that the expected number of cases was proportional to the length of the period in question, in other words, longer time periods were expected to have a higher number of cases. The normalisation of the expected number of cases assumed that the total number was equal to the total number of observations in the same period. The expected number of cases was calculated based on the annual population size of the study area and standardised by sex and age at diagnosis. The expected number of cases was assumed to be constant for periods of equal length within any calendar year. For “within-year” analyses, no adjustment was required, as it was assumed that the change in population size within the year was negligible. For the EPV between years analyses, it was not possible to take into consideration changes in the size of the population between years due to the lack of annual population data for the relevant age groups in the study area over the entire study period [4, 17].

## Results

**Fig. 1:**
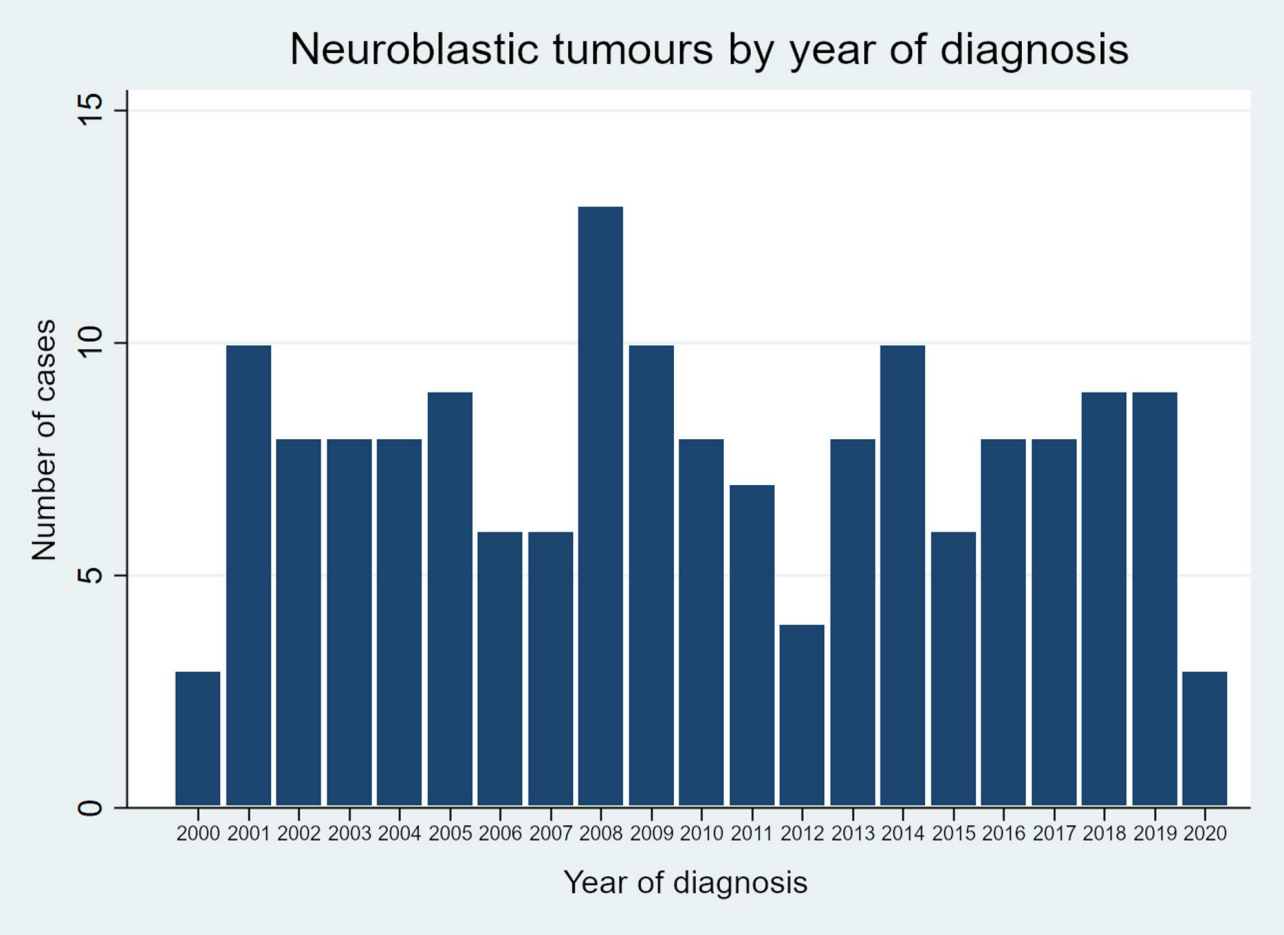
Number of cases per year of neuroblastic tumours at ages 0–24 years in Scotland. The number of cases by calendar year of diagnosis are shown

A total of 161 cases of neuroblastic tumours (148 cases of neuroblastoma and 13 cases of ganglioneuroblastoma) aged 0–24 years were diagnosed in Scotland during the period 2000–2020. Table 1 displays the number of cases by sex, age at diagnosis (due to small numbers two age groups were used, < 18 months and 18 months – 24 years) and period of diagnosis. The number of cases by year of diagnosis are presented in Fig. 1. Overall, there was evidence for long-term clustering of diagnosis, as shown by the significant EPV between years within the full study period (EPV = 9.13, SE = 0.22, *P* < 0.001). There was marginally significant evidence for short term extraneous variation between fortnights within months (EPV = 0.54, SE = 0.33, *P* = 0.053). However, there was no evidence for EPV between fortnights within quarters, within years or within the full study period. There was also no evidence for EPV between months or between quarters (Table 2).

**Table 1:** Characteristics of cases of neuroblastic tumours in the study population (based on cases diagnosed during 2000-2020 inclusive)

	Number of cases (%)			
Overall (0-24 years)	161 (100)			
Males	77 (47.8)			
Females	84 (52.2)			
Age at diagnosis:				
<18 months	55 (34.2)			
18 months – 4 years	70 (43.5)			
5 – 24 years	36 (22.4)			
Time period:				
2000-2005	46 (28.6)			
2006-2010	43 (26.7)			
2011-2015	35 (21.7)			
2016-2020	37 (23.0)			

	Mean	Median	Quartile 1	Quartile 3
Age at diagnosis (years)	3.44	2.40	0.88	4.38
Annual number of cases	7.67	8	6	9

**Table 2:** Analyses of temporal clustering of neuroblastic tumours at ages 0-24 years

Type of analysis	^β^1^ (SE)			
	*One-sided p value*			
	Within months	Within quarters	Within years	Within full period
Between fortnights	0.54 (0.33) *P* = 0.053	0.14 (0.11) *P* = 0.115	0.05 (0.07) *P* = 0.230	0.02 (0.06)* P* = 0.367
Between months		-0.05 (0.17) *P* = 0.607	-0.06 (0.10) *P* = 0.730	-0.09 (0.09) *P *= 0.845
Between quarters			-0.10 (0.19) *P* = 0.688	-0.16 (0.16)* P* = 0.842
Between years				**9.13 (0.22)*P < 0.001***

Statistically significant results have been emboldened

^1^^β is the one-step estimate of β, the extra-Poisson variation, calculated in the same way as originally described by Muirhead [4]

The separate analysis of cases diagnosed before 18 months of age showed that EPV was exhibited between months within quarters (EPV = 0.77, SE = 0.41, *P* = 0.044) and between years within the full study period (EPV = 8.03, SE = 0.23, *P* < 0.001). There was marginally significant evidence for EPV between months within years (EPV = 0.18, SE = 0.13, *P* = 0.092). There was no evidence for EPV between months within the full study period, between fortnights, or between quarters (Table 3).

**Table 3:** Analyses of temporal clustering of neuroblastic tumours by age-group

Type of analysis	^β^1^ (SE)			
	*One-sided p value*			
	Within months	Within quarters	Within years	Within full period
Ages < 18 months				
Between fortnights	-0.01 (0.71) *P* = 0.516	0.30 (0.26) *P* = 0.146	0.03 (0.09) *P* = 0.337	0.04 (0.06)* P* = 0.277
Between months		**0.77 (0.41)*P = 0.044***	0.18 (0.13) *P *= 0.092	0.08 (0.09)* P *= 0.192
Between quarters			-0.12 (0.25) *P* = 0.664	-0.10 (0.16)* P* = 0.720
Between years				**8.03 (0.23)** ***P< 0.001***
Ages 18 months to 24 years				
Between fortnights	**1.00 (0.47)** ***P = 0.012***	0.20 (0.14) *P* = 0.089	0.11 (0.07) *P* = 0.070	0.08 (0.06) *P* = 0.115
Between months		-0.06 (0.23) *P *= 0.597	-0.02 (0.11) *P* = 0.564	-0.04 (0.09) *P *= 0.674
Between quarters			0.15 (0.20) *P* = 0.227	-0.04 (0.16) *P* = 0.583
Between years				**9.24 (0.22)** ***P < 0.001***

Statistically significant results have been emboldened

^1^^β is the one-step estimate of β, the extra-Poisson variation, calculated in the same way as described by Muirhead [4]

The analysis of cases diagnosed at ages 18 months to 24 years found evidence of EPV between fortnights within months (EPV = 1.00, SE = 0.47, *P* = 0.012) and between years within the full study period (EPV = 9.24, SE = 0.22, *P* < 0.001). There was marginally significant evidence for EPV between fortnights within quarters (EPV = 0.20, SE = 0.14, *P* = 0.089) and, also, between fortnights within years (EPV = 0.11, SE = 0.07, *P* = 0.070). There was no evidence for EPV between fortnights within the full study period, between months, or between quarters (Table 3).

Separate analyses were conducted for males and females. For males, there was evidence for long-term variation in patterns of diagnosis, as shown by the significant EPV between years within the full study period (EPV = 8.10, SE = 0.22, *P* < 0.001). There was marginally significant evidence for short term extraneous variation between fortnights within months (EPV = 0.90, SE = 0.76, *P* = 0.059). However, there was no evidence for EPV between fortnights within quarters, within years or within the full study period. There was also no evidence for EPV between months or between quarters (Table 4). For females, there was evidence for long-term variation in patterns of diagnosis, as demonstrated by the significant EPV between years within the full study period (EPV = 8.99, SE = 0.23, *P* < 0.001). However, there was no evidence for EPV between fortnights, between months or between quarters (Table 4).

**Table 4:** Analyses of temporal clustering of neuroblastic tumours at ages 0-24 years, separately, for males and females

Type of analysis	^β^1^ (SE)			
	*One-sided p value*			
	Within months	Within quarters	Within years	Within full period
Males				
Between fortnights	0.90 (0.76) *P* = 0.059	0.03 (0.21) *P* = 0.405	-0.05 (0.08) *P* = 0.712	-0.02 (0.06)* P* = 0.529
Between months		-0.30 (0.32) *P* = 0.818	-0.21 (0.12) *P* = 0.972	-0.12 (0.09)* P *= 0.903
Between quarters			-0.22 (0.23) *P* = 0.834	-0.16 (0.16)* P* = 0.851
Between years				**8.10 (0.22)* P < 0.001***
Females				
Between fortnights	0.16 (0.60) *P* = 0.446	-0.08 (0.17) *P* = 0.659	-0.01 (0.08) *P* = 0.542	-0.02 (0.06) *P* = 0.657
Between months		-0.06 (0.16) *P* = 0.617	-0.07 (0.11) *P *= 0.723	-0.07 (0.09) *P *= 0.790
Between quarters			0.08 (0.22) *P* = 0.342	-0.06 (0.16) *P* = 0.617
Between years				**8.99 (0.23)** ***P < 0.001***

Statistically significant results have been emboldened

^1^ ^β is the one-step estimate of β, the extra-Poisson variation, calculated in the same way as described by Muirhead [4]

## Discussion

The findings show temporal clustering of neuroblastic tumours in those aged 0–24 years over longer time periods (between years). There was also an indication (although not formally statistically significant) of EPV, indicating clustering between fortnights (within months). For patients aged less than 18 months, there was evidence of temporal clustering between months and between years, and for ages 18 months to 24 years (this age group was used due to the small numbers of cases included in the study) temporal clustering was exhibited between fortnights and between years. For both males and females there was temporal clustering between years. For males only there was a suggestion of EPV between fortnights (not formally statistically significant).

The results from the present study contrast with two previous studies of temporal clustering of neuroblastic tumours. One study from northern England found evidence of temporal clustering between fortnights and between months, but no evidence of clustering between years [4]. Another from Ontario, Canada found temporal clustering only between years, but not between fortnights, months or quarters [17]. The findings from the present study which analysed data from Scotland contrasted with the other two regions. These results suggest the presence of EPV in Scotland for both shorter and longer temporal periods. The reasons for the differences between the three studies is unclear but could be linked to some aspect of geography or the socioeconomic context. Differences in socio-demographics may have led to differences in the nature and patterns of exposure to a postulated, but unknown agent implicated in the aetiology. There are marked differences in the population distribution and manifestation of deprivation between the study areas [20–23]. Scotland has a population of 5.4 million, of whom 12.9% are from ethnic minorities, living in both urban and highly rural areas [24]. Ontario has an ethnically diverse population of 14.7 million, of whom 11.3 million reside in urban areas. The province covers an area of 15,337 km^2^ [25]. With a total population of around 3.1 million, northern England comprises a mix of urban and rural areas with widely varying population densities and is ethnically homogeneous with low levels of the population from ethnic minorities [26].

These findings are consistent with the involvement of a putative transient aetiological agent. Examples of possible transient environmental exposures include pesticides, air-borne toxic exposures and infections [3, 4, 10]. Exposure to such agents is likely to vary by both temporal occurrence and geographical setting. Such heterogeneity in the possible patterns of exposure could have led to the differences between the manifestations of temporal clustering that have been observed in the present study and those seen in the two previously published analyses [4, 17].

Increased incidence of neuroblastoma has been noted in studies from China, 1990–2021 [27], Canada, 1992–2010 [28], Italy, 1967–2011 [29], the Netherlands, 1990–2017 [30], Ukraine and Belarus, 1990–2016 [31]. In contrast, decreases were noted in the USA, SEER 1990–2016, Slovenia and Cyprus [31]. The present study has demonstrated a decreasing trend in the incidence of neuroblastoma during the period 2000–2020. The population of Scotland aged 0–24 years was relatively stable, (1,538,542 in 2001, 1,548,819 in 2011 and 1,473,732 in 2022) [32]. However, there is a lack of annual data on population sizes and so this could not be taken into account in the between years analyses.

The finding of temporal clustering amongst children diagnosed with neuroblastic tumours in Scotland is distinct from other key types of clustering, namely space-time and spatial clustering. Previous studies from the whole of Great Britain did not find any evidence of space-time clustering amongst children diagnosed with neuroblastic tumours [33, 34]. It is noteworthy that a few other studies have identified some limited evidence in other countries of space-time clustering amongst neuroblastic tumours at diagnosis [reviewed in 35]. A previous study, again analysing data on neuroblastic tumours from the whole of Great Britain, did not find any evidence of spatial clustering [36].

In conclusion, these findings suggest that an aetiological agent that exhibits peaks and troughs in occurrence may play a role. The manifestation may differ between regions and countries. Larger scale studies are needed to confirm our findings. The findings should also be validated in a future extended Scottish dataset, but this would not be possible for quite some time.

## Data Availability

The data analysed during this study can be made available on reasonable request to the corresponding author, subject to relevant permissions for data sharing.
